# Role of anti-inflammatory compounds in human immunodeficiency virus-1 glycoprotein120-mediated brain inflammation

**DOI:** 10.1186/1742-2094-11-91

**Published:** 2014-05-16

**Authors:** Tamima Ashraf, Wenlei Jiang, Md Tozammel Hoque, Jeffrey Henderson, Chiping Wu, Reina Bendayan

**Affiliations:** 1 Department of Pharmaceutical Sciences, Leslie Dan Faculty of Pharmacy, University of Toronto, 144 College Street, Room 1001, Toronto, ON M5S 3 M2, Canada

**Keywords:** Chloroquine, HIV-1 brain inflammation, HIV-1 gp120, Inducible nitric oxide synthase, Maraviroc, Minocycline, Mitogen-activated protein kinase (MAPK), Pro-inflammatory cytokines, Simvastatin

## Abstract

**Background:**

Neuroinflammation is a common immune response associated with brain human immunodeficiency virus-1 (HIV-1) infection. Identifying therapeutic compounds that exhibit better brain permeability and can target signaling pathways involved in inflammation may benefit treatment of HIV-associated neurological complications. The objective of this study was to implement an *in vivo* model of brain inflammation by intracerebroventricular administration of the HIV-1 viral coat protein gp120 in rats and to examine anti-inflammatory properties of HIV adjuvant therapies such as minocycline, chloroquine and simvastatin.

**Methods:**

Male Wistar rats were administered a single dose of gp120_ADA_ (500 ng) daily for seven consecutive days, intracerebroventricularly, with or without prior intraperitoneal administration of minocycline, chloroquine or simvastatin. Maraviroc, a CCR5 antagonist, was administered intracerebroventricularly prior to gp120 administration for seven days as control. Real-time qPCR was used to assess gene expression of inflammatory markers in the frontal cortex, hippocampus and striatum. Interleukin-1β (IL-1β) and tumor necrosis factor-α (TNF-α) secretion in cerebrospinal fluid (CSF) was measured applying ELISA. Protein expression of mitogen-activated protein kinases (MAPKs) (extracellular signal-related kinase 1/2 (ERK1/2), c-Jun N-terminal kinases (JNKs) and P38 kinases (P38Ks)) was detected using immunoblot analysis. Student’s *t*-test and ANOVA were applied to determine statistical significance.

**Results:**

In gp120_ADA_-injected rats, mRNA transcripts of interleukin-1β (IL-1β) and inducible nitric oxide synthase (iNOS) were significantly elevated in the frontal cortex, striatum and hippocampus compared to saline or heat-inactivated gp120-injected controls. In CSF, a significant increase in TNF-α and IL-1β was detected. Maraviroc reduced upregulation of these markers suggesting that the interaction of R5-tropic gp120 to CCR5 chemokine receptor is critical for induction of an inflammatory response. Minocycline, chloroquine or simvastatin attenuated upregulation of IL-1β and iNOS transcripts in different brain regions. In CSF, minocycline suppressed TNF-α and IL-1β secretion, whereas chloroquine attenuated IL-1β secretion. In gp120-injected animals, activation of ERK1/2 and JNKs was observed in the hippocampus and ERK1/2 activation was significantly reduced by the anti-inflammatory agents.

**Conclusions:**

Our data demonstrate that anti-inflammatory compounds can completely or partially reverse gp120-associated brain inflammation through an interaction with MAPK signaling pathways and suggest their potential role in contributing towards the prevention and treatment of HIV-associated neurological complications.

## Background

Neurocognitive impairment remains highly prevalent in human immunodeficiency virus-1 (HIV-1) infected individuals due to persistence of viral replication and associated inflammation in the brain [1]. Since the initiation of highly active antiretroviral therapy (HAART), the development of HIV-associated dementia (HAD) in HIV-1 infected patients has significantly decreased. However, milder forms of HIV-associated neurocognitive disorders (HAND) are becoming more predominant in the post-HAART era, in part due to the longer life expectancy of infected individuals on treatment. Despite receiving treatment, up to 50% of HIV-1 infected patients continue to develop cognitive impairment, psychiatric illness and persistent fatigue [1].

In response to HIV-1, resident microglia in the brain become activated and release inflammatory mediators that further trigger the activation of neighboring microglia and astrocytes and signal recruitment of peripheral monocytes from the systemic circulation [2]. Studies have demonstrated that virus-infected cells or glial cells can release various neurotoxic factors (arachidonic acid, platelet activating factor, quinolinic acid, reactive oxygen species, nitric oxide, and glutamate) and a number of pro-inflammatory cytokines (tumor necrosis factor-α (TNF-α), interleukin-1β (IL-1β), IL-6, IL-8, IL-18, interferon α (IFNα), IFNγ) [2-4]. Irreversible neuronal injury and loss is often caused by inflammation and oxidative stress associated with these mediators present in the systemic circulation that crosses the blood–brain barrier and/or mediators secreted in the brain from glial cells. Detectable amounts of these cytokines in the cerebrospinal fluid (CSF) of patients with HIV-associated neurocognitive disorder have been reported [4]. *In vitro* studies in primary glial cell cultures also suggest that shed viral proteins (such as glycoprotein120 (gp120), transactivator of transcription (tat), and viral protein R (vpr)) can induce secretion of pro-inflammatory cytokines. For example, previous work from our laboratory has demonstrated that R5-tropic gp120 can mediate secretion of pro-inflammatory cytokines (TNF-α, IL-1β and IL-6) by interacting with CCR5 chemokine receptor in primary cultures of human or rodent astrocytes [5,6]. HIV-1 proteins (such as gp120 and tat) can also downregulate tight junction proteins expressed in brain microvessel endothelial cells which may facilitate the entry of the virus into the brain parenchyma [7].

The treatment of brain HIV-1 infection remains challenging partly due to poor permeability of antiretroviral drugs across the blood-brain barrier and into glial cells. One possible mechanism for the low brain permeability of these drugs is the functional expression of ATP-dependent, membrane-associated efflux transporters known as ATP-binding cassette (ABC) transporters (P-glycoprotein (P-gp); multidrug resistance proteins (MRPs) and breast cancer resistance protein (Bcrp)) at the blood-brain barrier and in brain parenchyma [8]. Several *in vivo* and *in vitro* studies have examined the role of these transporters in reducing the permeability of antiretroviral drugs into brain cellular compartments [8,9]. For example, administration of P-gp specific inhibitor zosuquidar in macaques resulted in significant brain accumulation of nelfinavir [9]. Evidence in the literature suggests that functional expression of these transporters in the brain is altered during HIV-1 infection. Langford *et al.* reported increased P-gp immunoreactivity in glial cells in brain autopsy tissues from patients with HIV-encephalitis (HIVE), [10] whereas, Persidsky *et al.* reported a decreased P-gp expression in tissues obtained from HIVE patients and from the severe combined immunodeficiency (SCID) mice model of HIVE [11]. Furthermore, shed viral proteins (such as gp120 and Tat) and secreted pro-inflammatory cytokines during HIV-1 infection are also known to alter the expression of drug efflux transporters. Our previous work in rodent and human astrocytes suggests that gp120 can significantly downregulate P-gp functional expression [5]. We have also observed an increase in Mrp1 functional expression in response to gp120 in primary cultures of rat astrocytes [3,12]. However, whether gp120 can modulate the expression of these transporters *in vivo* in a similar manner is yet to be characterized.

Several classes of antiretrovirals have been reported to poorly permeate into the brain, whereas better permeable antiretroviral drugs can be associated with adverse side effects including neurotoxicity [13]. Due to the complexities associated with the treatment of brain HIV-1 infection and HIV-associated chronic secretion of inflammatory mediators, much interest has risen in identifying potential adjuvant therapies that can be used along with antiretroviral drugs. For example, minocycline, a second generation tetracycline derivative, has been considered as a potential candidate due to its versatile role in neuroprotection in different brain disease models [14,15]. Chloroquine, an antimalarial drug and simvastatin, a 3-hydroxy-3-methylglutaryl coenzyme A reductase inhibitor, have also demonstrated an anti-inflammatory and anti-HIV effect at non-toxic concentrations *in vitro* or *in vivo *[16-19]. Furthermore, minocycline, chloroquine and simvastatin can inhibit mitogen-activated protein kinase (MAPK) signaling pathways (extracellular signal-related kinase 1/2 (ERK1/2), c-Jun N-terminal kinases (JNKs) and P38 kinases (P38Ks)) involved in generating inflammatory responses [20]. In a clinical trial minocycline treatment was found to be unsuccessful in improving the neurocognitive outcome in patients with cognitive impairment [21]. On the contrary, in a simian immunodeficiency virus (SIV) model early administration of minocycline was reported to be effective against striatal dopaminergic system dysfunction, suggesting that timely treatment initiation may have an effect on minocycline efficacy [15]. Based on these observations, we propose that early administration of these anti-inflammatory compounds may have the potential to reverse HIV-associated inflammatory response.

The objective of the present work was to implement an *in vivo* model of HIV-associated inflammation by intracerebroventricular (ICV) administration of viral coat protein gp120 and to investigate the regulation of drug transporters, tight junction proteins, signaling pathways and the potential anti-inflammatory effects of chloroquine, minocycline and simvastatin.

## Materials and methods

### Materials

HIV-1_ADA_ gp120 full-length protein (subtype B; R5-tropic) and maraviroc were obtained from Immunodiagnostic Inc (Woburn, Massachusetts, United States) and the National Institutes of Health AIDS Research and Reference Reagent program (Bethesda, Maryland, United States), respectively. ERK1/2 inhibitor U0126 and simvastatin were purchased from Cell Signaling (Whitby, Ontario, Canada) and Toronto Research Chemicals (Toronto, Ontario, Canada), respectively. Chloroquine, minocycline, JNK inhibitor SP600125, murine monoclonal AC-40 antibody against actin, rabbit polyclonal antibody against glial fibrillary acidic protein (GFAP) and horseradish peroxidase (HRP) conjugated secondary antibodies (anti-mouse, anti-rabbit and anti-rat) were obtained from Sigma-Aldrich (Oakville, Ontario, Canada). Alexa fluor 488 donkey anti-rabbit, anti-occludin, anti-zo1 and anti-claudin5 antibody, TRIzol™, DNAse I and Prolong gold anti-fade reagent with 4',6-diamidino-2-phenylindole (DAPI) were purchased from Invitrogen (Carlsbad, California, United States). Tissue-Tek cryo-optimum cutting temperature (OCT) formulation was purchased from Thermo Fisher Scientific (Waltham, Massachusetts, United States). ERK1/2, phospho-ERK1/2, JNK, phospho-JNK, P38K and phospho-P38K antibodies were obtained from Cell Signaling (Whitby, Ontario, Canada). Murine monoclonal C219 antibody against P-gp, rat monoclonal MRP1 antibody and anti-BCRP (rat monoclonal) antibody were purchased from ID labs (London, Ontario, Canada), Kamiya Biomedical Company (Seattle, Washington, United States) and Abcam Inc (Boston, Massachusetts, United States), respectively. Enzyme linked immunoabsorbant assay (ELISA) kits were obtained from R&D Systems (Minneapolis, Minnesota, United States). Guide cannula, dummy cannula, screws and dental cement for stereotaxic surgery were purchased from HRS Scientific (Montreal, Quebec, Canada). High capacity reverse transcriptase cDNA synthesis kit and SYBR Green Fastmix were obtained from Applied Biosystems (Foster City, California, United States) and Quanta Biosciences Inc (Gaithersburg, Maryland, United States), respectively.

### Animals

This study was approved by the University of Toronto Animal Care Committee, Toronto, Ontario, Canada. Adult Wistar male rats (weighing approximately 250 grams; 12 weeks of age) were purchased from Charles River Laboratories (St. Constant, Quebec, Canada). Animals were housed with rodent chow and water ad libitum on a 12 hour light-dark cycle. All procedures were carried out in accordance with the guidelines of University of Toronto Animal Care Committee and the Canadian Council on Animal Care. The rats were randomly assigned to nine different groups: (1) wild-type (no surgery), (2) control (saline), (3) heat-inactivated gp120, (4) gp120, (5) maraviroc + gp120, (6) chloroquine + gp120, (7) minocycline + gp120, (8) simvastatin + gp120, (8) JNK inhibitor SP600125 + gp120, and (9) ERK1/2 inhibitor U0126 + gp120.

### Animal surgery and ICV administration of gp120/maraviroc/signaling pathway inhibitors

Animals were anaesthetized with isoflurane (2 to 3%). The implantation of the guide cannula was performed according to previously published protocols using the following stereotaxic coordinates: 1 mm posterior to bregma, 1.5 mm lateral from midline and 3.5 mm ventral from the surface of the skull according to the Atlas of Paxinos and Watson [22,23]. Ketoprofen (5 mg/kg) was administered subcutaneously during surgery for pain relief. Animals were allowed to recover for seven days after surgery. Following recovery, animals were administered a single dose of (100 ng or 300 ng or 500 ng) gp120_ADA_ (R5-tropic; clade B) daily for seven consecutive days ICV using a 5 μl Hamilton syringe. gp120 was diluted into a sterile normal saline solution and administered in a volume of 2 μl (such as 250 ng/μl) over 2 minute period. Control animals received equal amount of saline. Heat-inactivated gp120 was also administered for seven days as an additional control group. Maraviroc, a selective CCR5 antagonist, was administered 30 minutes prior to gp120 administration for seven days (4 μl/4 minutes; 250 ng/μl in 1% dimethyl sulfoxide (DMSO)). ERK1/2 inhibitor U0126 (7 μg) and JNK inhibitor SP600125 (10 μg) were administered ICV prior to gp120 administration at the same rate (4 μl/4 minutes; dissolved in 1.5% DMSO).

### Intraperitoneal administration of minocycline, chloroquine and simvastatin

Minocycline (50 mg/kg loading dose followed by 25 mg/kg/day) or chloroquine (25 mg/kg/day) or simvastatin (1 mg/kg) was administered daily for seven consecutive days by a single intraperitoneal injection 30 minutes prior to ICV gp120 administration. Minocycline and chloroquine were dissolved in sterile water and simvastatin was dissolved in a 1% DMSO solution.

### Brain tissue, brain capillary isolation and cerebrospinal fluid collection

Twenty four hours after the last injection, animals were anaesthetized and CSF samples were collected by cisterna magna puncture according to previously published protocol [24]. Subsequently, anaesthetized animals were perfused through the left ventricle of the heart with 200 ml of saline solution and whole rat brains were harvested. For real-time qPCR and immunoblot analysis, brains were dissected on ice to isolate frontal cortex, hippocampus and striatum from both hemispheres. Samples were flash frozen in liquid nitrogen and kept at -80°C. Capillaries were also isolated from saline and gp120-injected rat brains following previously published protocol [25]. For immunohistochemical analysis, saline perfusion was followed by 180 ml of paraformaldehyde (4%) dissolved in phosphate-buffered saline (PBS) perfusion before collecting whole brains. These samples were stored in 50 ml paraformaldehyde (4%) at 4°C.

### Quantitative real-time PCR

Real-time qPCR was applied to determine the gene expression of inflammatory markers according to previously described protocols followed by our laboratory [26]. Briefly, total RNA was extracted from brain regions using TRIzol™ (Invitrogen, Carlsbad, California, United States) reagent and the high capacity cDNA reverse transcriptase kit (Applied Biosystems, Foster City, California, United States) was used to synthesize first-strand cDNA. Prixmer pairs for the rat interleukin-1β (IL-1β) gene (5-CTCAACTGTGAAATAGCAGCTTTC–3 and 5 GGACAGCCCAAGTCAAGG–3), rat tumor necrosis factor-α (TNF-α) (5-TCTTCTCATTCCTGCTCGTG–3 and 5-GATGAGAGGGAGCCCATTT–3), rat inducible nitric oxide synthase (iNOS) (5-CCAAGGTGACCTGAAAGAGG–3 and 5 TTGATGCTTGTGACTCTTAGGG–3), rat IL-6 (5-CTTCACAAGTCGGAGGCTTAAT–3 and 5-ACAGTGCATCATCGCTGTTC–3) and the rat Cyclophilin B gene (housekeeping gene; 5-GGAGATGGCACAGGAGGAA-3 and 5-GCCCGTAGTGCTTCAGCTT-3) were designed using Primer Express 3 software (Applied Biosystems, Foster City, California, United States)) and were validated for specificity and efficacy using lipopolysaccharide-treated rat astrocyte samples. Quantitative PCR was performed using SYBR Green Master Mix (Quanta Biosciences Inc, Gaithersburg, Maryland, United States) using an Eppendorf Real-time PCR System (Eppendorf Canada, Mississauga, Ontario, Canada) at 25°C for 10 minutes, followed by 37°C for 120 minutes, and 85°C for 5 minutes. A relative quantification was performed by comparing the threshold cycle values of samples with serially diluted standards. Expression levels were normalized to housekeeping gene Cyclophilin B.

### Enzyme linked immunoabsorbant assay (ELISA)

Commercially available colorimetric sandwich ELISA kits for rat IL-1β and TNF-α were used to detect the secretion of these cytokines in CSF in the presence or absence of maraviroc, chloroquine, minocycline and simvastatin. CSF collected from control (no surgery) and ICV heat-inactivated gp120-administered animals was analyzed as an additional control group. The assays were performed according to the manufacturer’s instruction with slight modifications as previously described in our laboratory. Standard curves were generated using appropriate rat cytokines and absorbance read at 450 nm was converted to pg/ml. We confirmed the minimal detection level to be 2.5 pg/ml and 1.5 pg/ml for IL-1β and TNF-α, respectively.

### Immunoblot analysis

Immunoblotting was performed as described previously in our laboratory [5]. Tissue and capillary homogenates were prepared using a lysis buffer (1% (v/v) NP-40 in 20 mM tris, 150 mM NaCl, 5 mM ethylenediaminetetraacetic acid (EDTA) at pH 7.5 containing 1 mM phenylmethanesulfonyl fluoride and 0.1% (v/v) protease inhibitor cocktail). Tissues were sonicated for 10 seconds and centrifuged at 20,000 g for 10 minutes at 4°C to remove cellular debris. Protein concentrations of tissue or capillary homogenates were determined using Bradford’s protein assay (Bio-rad laboratories, California, United States). Total protein (1 μg, 5 μg to 50 μg) were separated on 7 to 12% sodium dodecyl sulfate polyacrylamide gel electrophoresis (SDS-PAGE) and transferred onto a polyvinylidene difluoride membrane. After blocking with 5% skim milk for one hour, the membrane was probed for protein of interest with primary antibody (anti-P-gp (C219), 1:500; Mrp1, 1:500; Bcrp, 1:500; β-actin, 1:2000; ZO-1, 1:500; occludin, 1:500; claudin5, 1:250; GFAP, 1:1000; iNOS, 1:500; ERK1/2,1:250; phospho-ERK1/2, 1:100; JNK, 1:250; phospho-JNK, 1:100; P38K, 1:250; phospho-P38K, 1:100). β-Actin was used as loading control. HRP-conjugated secondary antibody was added after washes in tris-buffered saline with Tween. After further washing, bands were detected using enhanced chemiluminescent reagent (Thermo Fischer Scientific, Ontario, Canada). Densitometric analysis was performed in AlphaDigiDoc RT2 software (Alpha Innotech, San Leandro, California, United States) to quantify relative protein expression. The graphs represent relative density of the bands of interest normalized to corresponding β-actin.

### Immunohistochemistry

Immunohistochemistry was performed according to previously published protocol [27]. Briefly, paraformaldehyde-fixed samples were transferred to a 30% sucrose solution and kept overnight at 4°C before mounting with OCT compound. Coronal cryostat sections (25 μm) were prepared at Murine Imaging and Histology Facility (Faculty of Pharmacy, University of Toronto, Toronto, Ontario, Canada). Sections were incubated in blocking buffer (5% goat serum and 0.2% Triton X-100 in 0.1 M PBS) for one hour followed by overnight incubation with primary antibody at 4°C (Anti-GFAP, 1:200). Following incubation, sections were washed thoroughly in PBS and incubated for two hours in Alexa-Fluor conjugated secondary antibody (1:200). After further washing, coverslips were mounted onto the specimen using a prolong gold anti-fade mounting medium with DAPI. Specimens were examined using a Nikon E1000 fluorescent microscope (Nikon Corp., Mississauga, Canada). Images were captured using the software SimplePCI (Compix, Inc., Imaging Systems, Sewickley, Pennsylvania, United States).

### Data analysis

Student’s *t*-test was used to determine statistical significance between two groups. Multiple comparisons were performed using analysis of variance (ANOVA) and Bonferroni’s *post-hoc* analysis. A *P* value less than 0.05 was considered to be statistically significant. Data were analyzed using GraphPad Prism software (San Diego, California, United States). Samples collected from three to twelve animals per group were used.

## Results

### gp120-mediated inflammatory response in the brain

**Figure 1:**
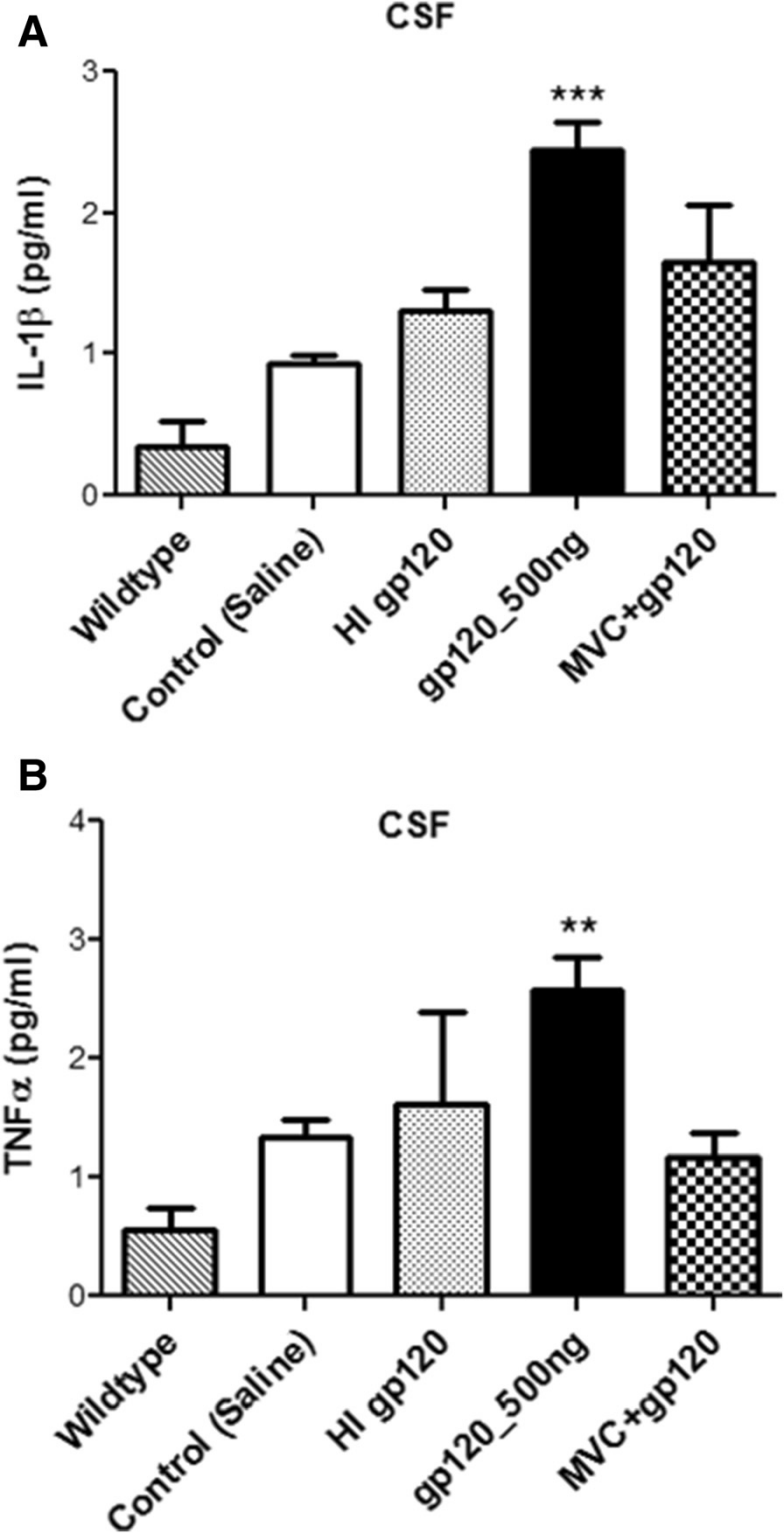
**Effect of gp120 on the mRNA levels of (A) IL-1β, (B) iNOS and (C) TNF-α in different brain regions of ICV-administered gp120 (500 ng) rats along with a CCR5 antagonist, maraviroc (MVC).** Wild-type (no surgery), control (saline) and heat-inactivated (HI) gp120-injected animals were also analyzed. Results are expressed as mean ± SEM (n = 10 for saline and gp120 groups; n = 3 for wild-type, HI gp120 and maraviroc-treated groups). Asterisks represent data points significantly different from saline-administered animals (***P <0.001; **P <0.01; *P <0.05). (gp120 = glycoprotein120; IL-1β = interleukin-1β; iNOS = inducible nitric oxide synthase; TNFα = tumor necrosis factor-α; ICV = intracerebroventricular; MVC = Maraviroc; HI = heat inactivated).

Previous studies have demonstrated that ICV or region-specific administration of different strains of gp120 from 100 ng to 500 ng into rodents can induce an inflammatory response [28,29]. In our hands, administration of 100 ng to 300 ng HIV-1 gp120_ADA_ daily for seven days in adult male Wistar rats did not induce a significant inflammatory response in brain tissue (data not shown). Therefore, a dose of 500 ng was chosen. At this dose, we observed a significant upregulation of IL-1β, iNOS and TNF-α in different brain regions. Compared to saline-administered animals, in gp120 (500 ng)-injected rats, transcripts of IL-1β, iNOS and TNF-α were significantly elevated in the frontal cortex, whereas, elevated IL-1β and iNOS mRNA were observed in the hippocampus and striatum (Figure 1A-C). No significant change was observed in IL-6 transcript levels (data not shown). Heat-inactivated gp120 did not induce any changes in inflammatory markers and remained comparable to saline treatment (Figure 1A-C). ANOVA analysis performed for different regions in Figure 1 did not show a significant difference of IL-1β, iNOS and TNF-α transcripts between wild-type animals (no surgery) and saline-treated animals (Figure 1A-C).

**Figure 2:**
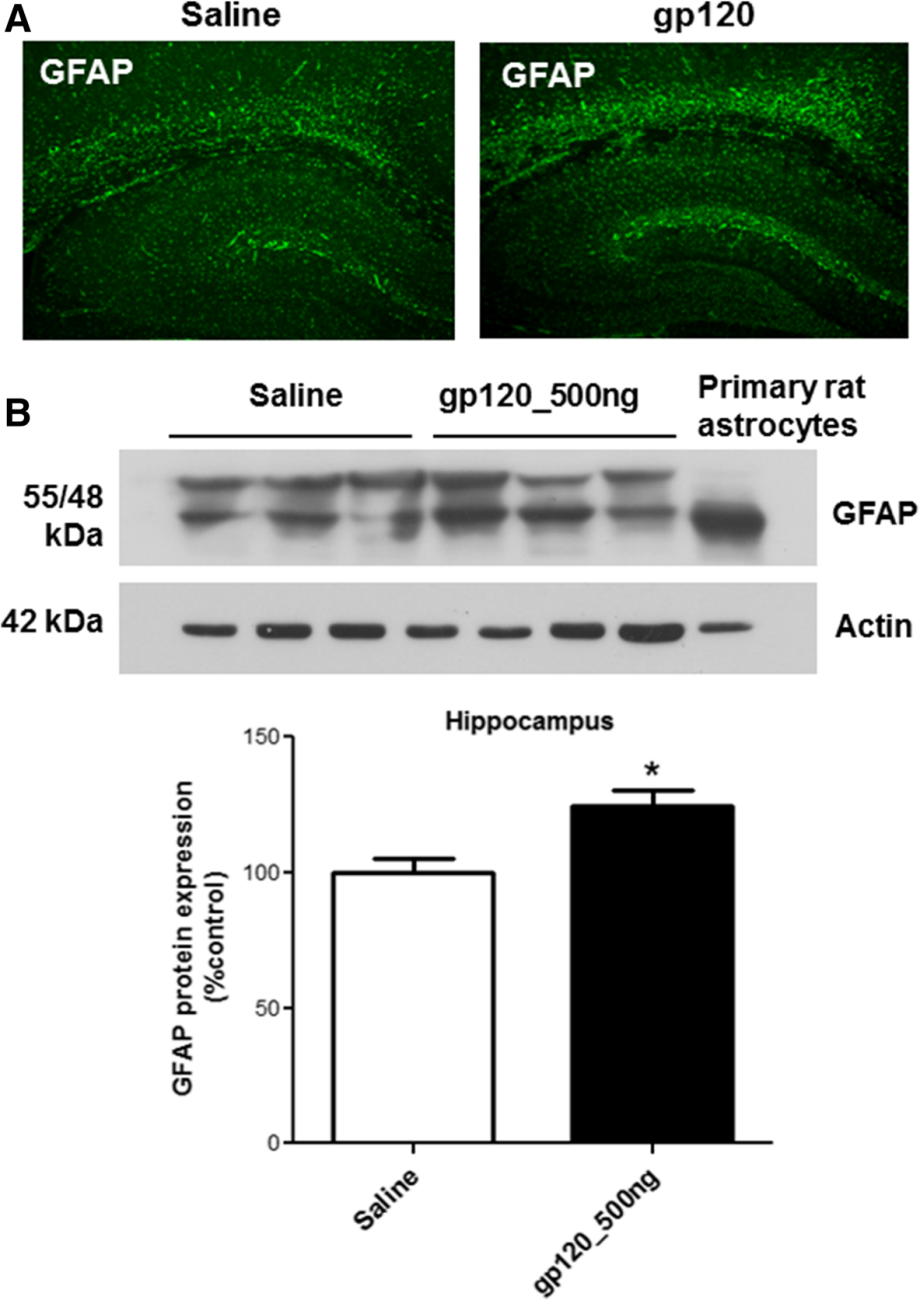
**ELISA analysis of (A) IL-1β and (B) TNF-α secretion in the CSF of gp120 (500 ng) administered rats in the presence of maraviroc (MVC).** CSF collected from saline- and heat-injected animals were used as control. Results are expressed as mean ± SEM (n = 10 for saline and gp120 groups; n ≥3 for wild-type, HI gp120 and maraviroc-treated groups). Asterisks represent data points significantly different from saline-administered animals (***P <0.001; **P <0.01). (ELISA = Enzyme linked immunoabsorbant assay; IL-1β = interleukin-1β; TNFα = tumor necrosis factor-α; CSF = cerebrospinal fluid; gp120 = glycoprotein120; MVC = Maraviroc; HI = heat inactivated).

Our previous work *in vitro* suggested that R5-tropic gp120 could interact with CCR5 chemokine receptors resulting in pro-inflammatory cytokine secretion [5]. In order to demonstrate that the inflammatory response observed in gp120-administered animals is mediated by the specific interaction of gp120 with CCR5 chemokine receptor *in vivo*, we administered maraviroc, a CCR5 antagonist ICV prior to gp120 administration in rodents. Administration of maraviroc (1 μg) resulted in a downregulation of IL-1β, iNOS and TNF-α transcript levels in different brain regions when compared to only gp120-treated animals (Figure 1A-C).At the protein level, a significant increase in TNF-α and IL-1β was also detected in CSF samples collected from gp120-administered animals (Figure 2A and B). Consistent with the qPCR data, maraviroc treatment attenuated the gp120-mediated secretion of cytokines. Also, heat-inactivated gp120-mediated secretion of IL-1β and TNF-α was found to not be significantly different from saline-treated animals (Figure 2A and B).

### gp120-mediated activation of astrocytes

**Figure 3:**
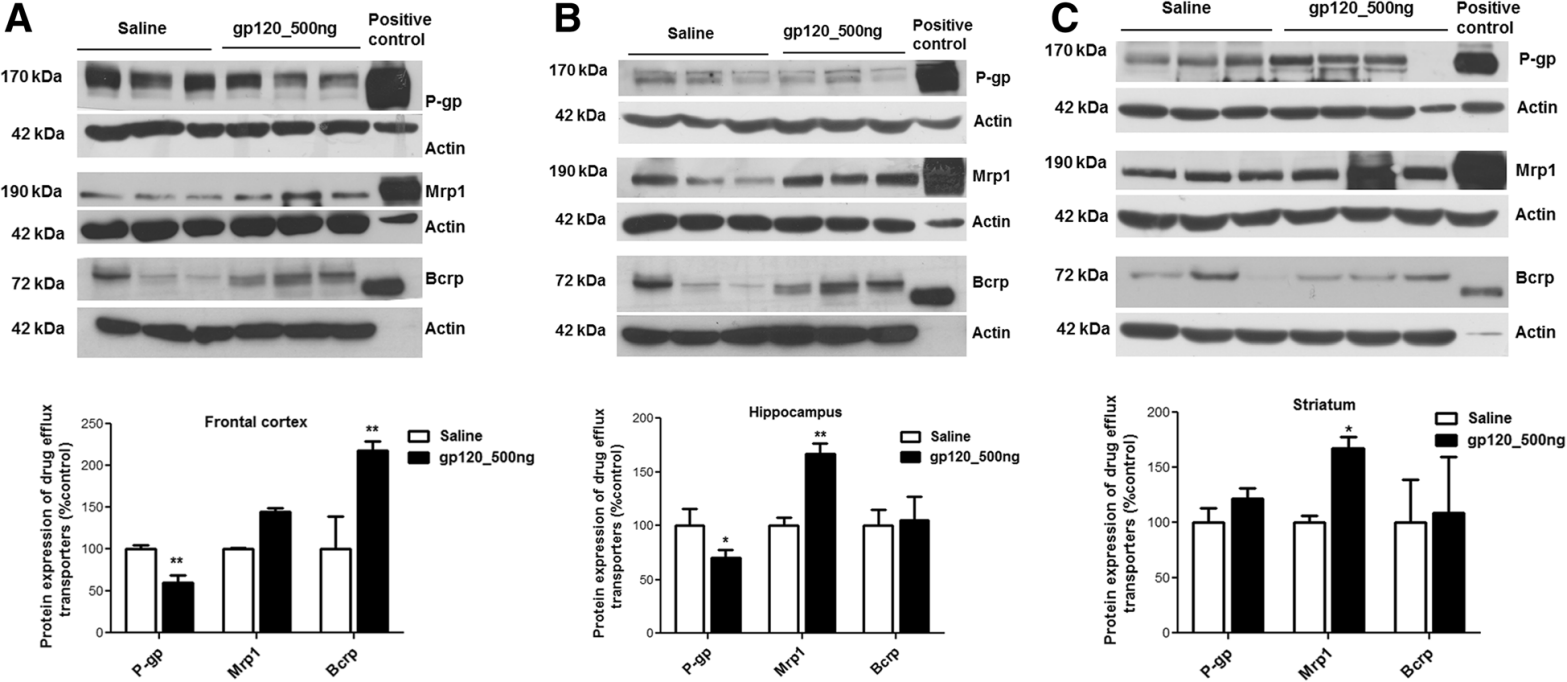
**GFAP expression in the hippocampus of ICV-administered gp120 rats. (A)** Immunohistochemical, **(B)** immunoblotting (upper panel) and densitometric analysis (lower panel) of GFAP in the hippocampus of ICV-administered gp120 rats are compared to saline-treated animals. A representative blot is shown. Results are expressed as mean ± SEM. For immunoblotting, samples obtained from five different animals were used per group. Asterisk represents a data point significantly different from saline-administered animals (*P <0.05). (GFAP = glial fibrillary acidic protein; ICV = intracerebroventricular; gp120 = glycoprotein120).

ICV administration of gp120 has previously been shown to result in astrocyte activation *in vivo* as demonstrated by an increase in GFAP expression, a cellular marker for astrocyte [29]. Therefore, in our model we investigated expression of GFAP using immunohistochemistry (Figure 3A). In gp120-administered animals, we observed an increase in GFAP staining in the hippocampus. This was further confirmed by immunoblotting analysis (Figure 3B).

### Effect of gp120 on tight junction proteins and drug efflux transporters in brain capillaries

Previous studies have reported a breakdown of the blood-brain barrier in models of brain HIV-1 infection *in vitro* and *in vivo *[11]. In order to determine the integrity of the blood-brain barrier in our *in vivo* model, we examined the expression of tight junction proteins (occludin, ZO-1, claudin5) as well as drug efflux transporters (P-gp, Mrp1 and Bcrp) in brain capillaries isolated from gp120-treated animals. We did not observe any significant changes in ZO-1, occludin, claudin5, P-gp, Mrp1 and Bcrp protein expression in brain capillaries isolated from saline or gp120-administered rats (data not shown).

### Effect of gp120 on drug efflux transporter expression in brain regions

**Figure 4:**
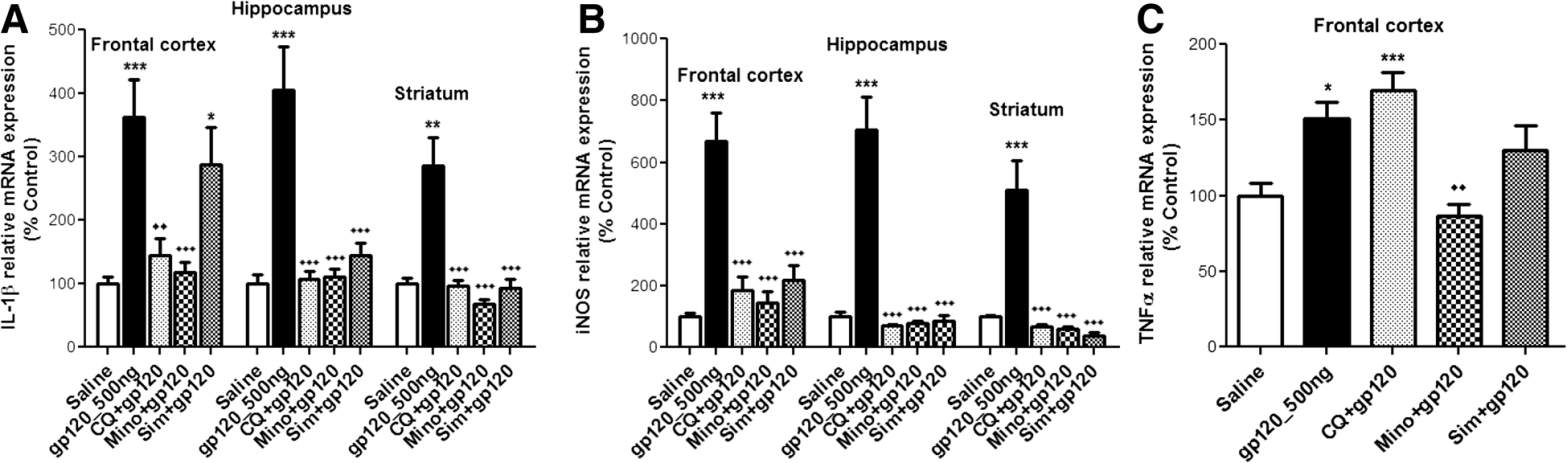
**Immunoblot (upper panel) and densitometric analysis (lower panel) of drug efflux transporters (P-gp, Mrp1 and Bcrp) in (A) frontal cortex, (B) hippocampus and (C) striatum of ICV-administered gp120 rats.** Transporter over-expressing cell lines were used as positive controls. Representative blots are shown. Results are expressed as mean ± SEM. Samples obtained from six different animals were used per group. Asterisks represent data points significantly different from saline-administered animals (**P <0.01; * *P* <0.05). (P-gp = P-glycoprotein; Mrp1 = multidrug resistance protein 1; Bcrp = breast cancer resistance protein; ICV = intracerebroventricular; gp120 = glycoprotein120).

We have previously shown *in vitro* that gp120 exposure can significantly decrease P-gp protein expression and increase Mrp1 protein expression in primary cultures of rat astrocytes and/or human fetal astrocytes. However, it was unknown if gp120 could alter the expression of these transporters *in vivo*. Applying immunoblot analysis, we detected significant changes in P-gp, Mrp1 and Bcrp protein expression in different brain regions of gp120-treated animals (Figure 4). gp120 administration resulted in a significant decrease in P-gp protein expression in the frontal cortex and hippocampus (40 and 30%, respectively) (Figure 4A and B). In the striatum of gp120-treated animals, we did not detect any significant changes in P-gp protein expression compared to saline-administered animals (Figure 4C). A significant upregulation of Mrp1 was observed in hippocampus and striatum (67 and 68%, respectively) (Figure 4B and C), whereas, no significant change was detected in the frontal cortex (Figure 4A). Bcrp, another drug efflux transporter, was found to be upregulated in the frontal cortex (118% compared to saline) (Figure 4A) but not in the hippocampus and striatum (Figure 4B and C).

### Anti-inflammatory effects of chloroquine, minocycline and simvastatin

**Figure 5:**
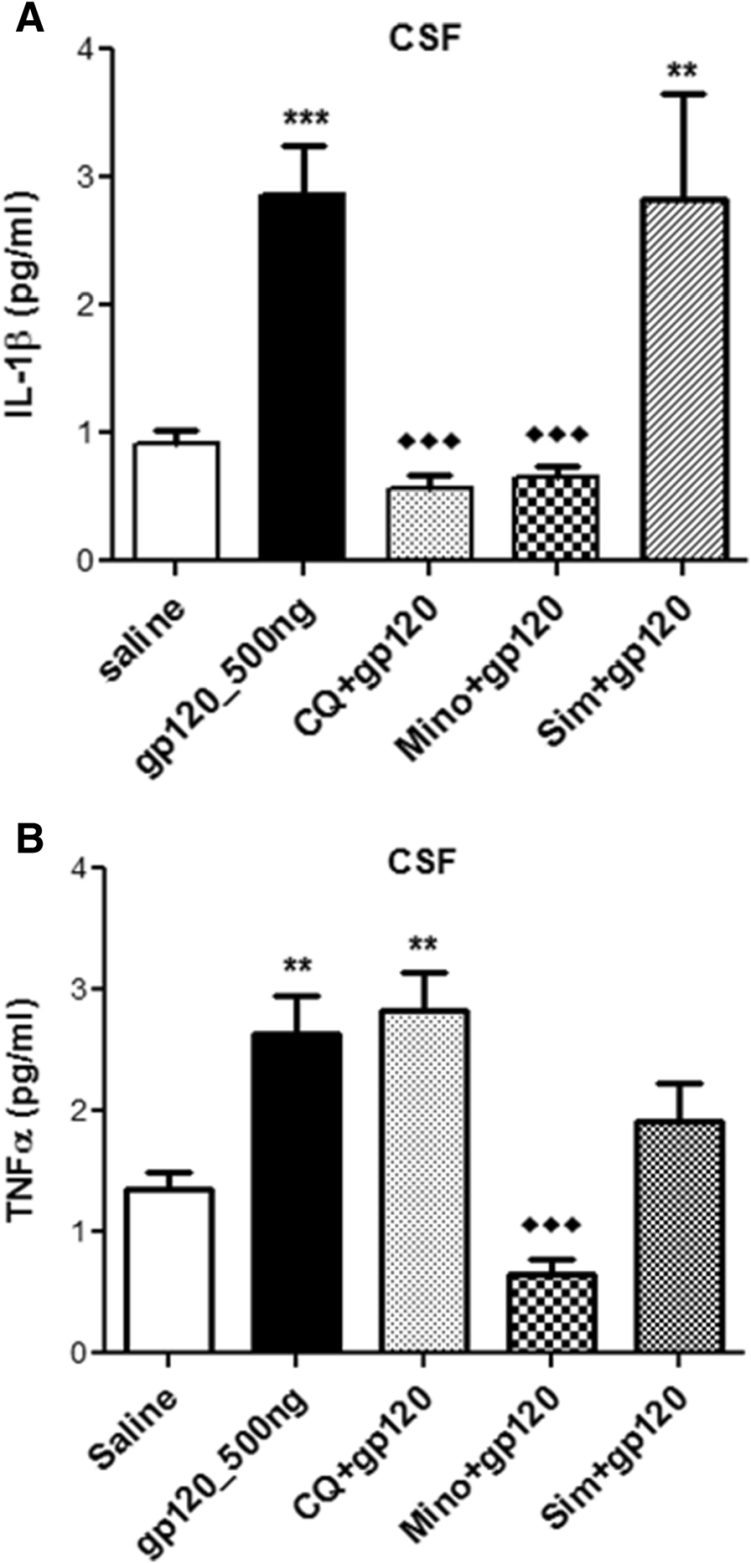
**Effect of gp120 on the mRNA levels of (A) IL-1β, (B) iNOS and (C) TNF-α in different brain regions of ICV-administered gp120 rats receiving simultaneous intraperitoneal injection of chloroquine or minocycline or simvastatin.** Saline-injected animals were used as control. Results are expressed as mean ± SEM. Samples obtained from at least 10 different animals were used per group. Asterisks represent data points significantly different from saline-administered animals (***P <0.001; **P <0.01; *P <0.05). Diamonds represent data points significantly different from gp120 treatment (♦♦♦*P* <0.001; ♦♦*P* <0.01). (gp120 = glycoprotein120; IL-1β = interleukin-1β; iNOS = inducible nitric oxide synthase; TNFα = tumor necrosis factor-α; ICV = intracerebroventricular; CQ = chloroquine; Mino = minocycline; Sim = simvastatin).

**Figure 6:**
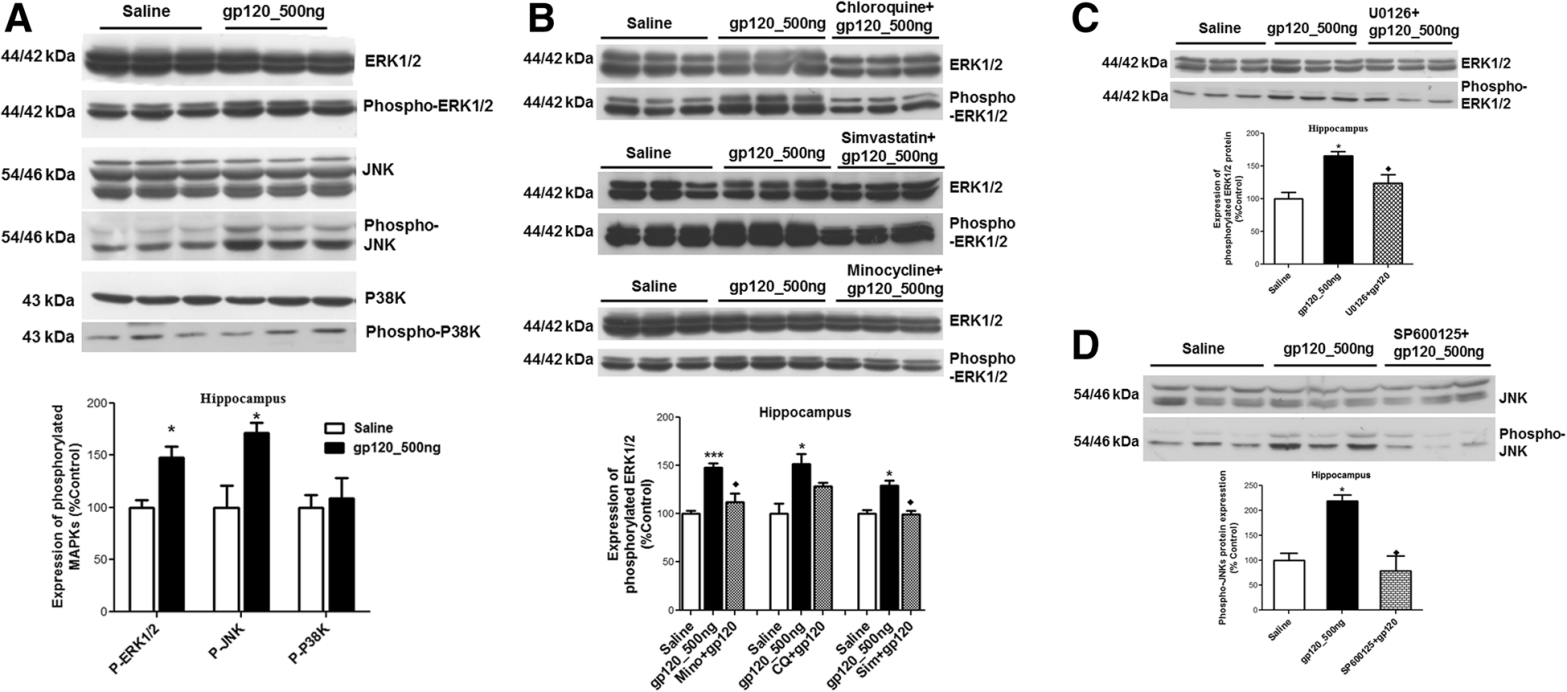
**ELISA analysis of (A) IL-1β and (B) TNF-α secretion in the CSF of gp120 (500 ng) administered rats receiving simultaneous intraperitoneal injection of chloroquine or minocycline or simvastatin.** CSF collected from saline-injected animals was used as a control. Results are expressed as mean ± SEM. Samples obtained from at least 10 different animals were used per group. Asterisks represent data points significantly different from saline-administered animals (***P <0.001; **P <0.01). Diamonds represent data points significantly different from gp120 treatment (♦♦♦*P* <0.001). (ELISA = Enzyme linked immunoabsorbant assay; IL-1β = interleukin 1β; TNFα = tumor necrosis factorα; CSF = cerebrospinal fluid; gp120 = glycoprotein120; CQ = chloroquine; Mino = minocycline; Sim = simvastatin).

Animals were administered gp120 (500 ng) ICV with or without prior intraperitoneal administration of minocycline or chloroquine or simvastatin in order to assess their potential anti-inflammatory effect. Administration of minocycline or chloroquine partially or completely suppressed the gp120-induced upregulation of the inflammatory markers examined (Figure 5). In particular, administration of minocycline or chloroquine completely attenuated gp120-mediated upregulation of IL-1β and iNOS transcripts in all three brain regions (Figure 5A-C).Minocycline was also effective in suppressing TNF-α transcripts in brain tissues and both TNF-α and IL-1β secretion in the CSF of gp120-administered animals, whereas chloroquine only attenuated IL-1β secretion (Figure 6A and B). Simvastatin attenuated both IL-1β and iNOS transcripts in the hippocampus and striatum (Figure 5A and B), but only suppressed iNOS in the frontal cortex (Figure 5B). At the protein level, however, simvastatin could not significantly suppress gp120-mediated IL-1β and TNF-α secretion in the CSF (Figure 6A and B).

### gp120-mediated activation of signaling pathways

**Figure 7:**
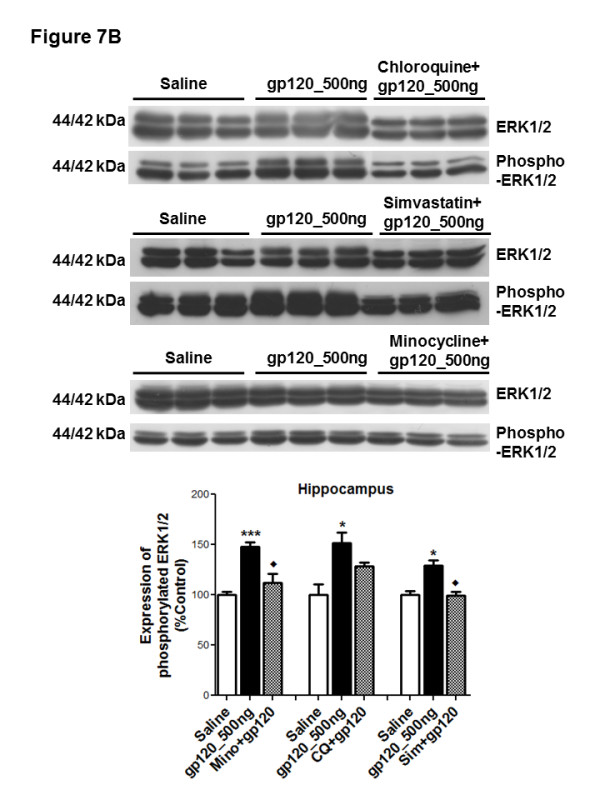
**Protein (upper panel) and densitometric analysis (lower panel) of (A) ERK1/2 and phospho-ERK1/2; JNK and phospho-JNK; P38K and phospho-P38K, (B) ERK1/2 and phospho-ERK1/2 in the presence of chloroquine, minocycline and simvastatin, (C) ERK1/2 and phospho-ERK1/2 in the presence of ERK1/2 inhibitor U0126; (D) JNK and phospho-JNK in the presence of SP600125 in the hippocampal tissue of gp120-administered rats.** Representative blots are shown. Tissue collected from saline-administered animals was used as control. Results are expressed as mean ± SEM. Samples obtained from three to six different animals per group were used. Asterisks represent data points significantly different from saline-administered animals (***P <0.001; *P <0.05). Diamonds represent data points significantly different from gp120-treatment (♦*P* <0.05). (ERK1/2 = extracellular signal-related kinase 1/2; p-ERK1/2 = phospho- ERK1/2; JNKs = c-Jun N-terminal kinases; p-JNKs = phospho-JNKs; P38Ks = P38 kinases; p-P38Ks = phosphor-p38Ks; gp120 = glycoprotein120).

The three main subfamilies of the MAPK pathway (ERK1/2, JNK and P38K) are known to be actively involved in regulating cytokine secretion [30]. Therefore, we investigated the activation of ERK1/2, JNK and P38K in different brain regions of gp120-administered animals. In the hippocampus, we observed phosphorylation of ERK1/2 and JNKs (Figure 7A), however, we did not detect significant changes in ERK1/2, JNK or P38K phosphorylation in the frontal cortex and striatum (data not shown). Administration of minocycline and simvastatin significantly reduced ERK1/2 phosphorylation, whereas the effect of chloroquine was not found to be significant (Figure 7B). gp120-mediated JNK phosphorylation was found to not be affected despite treatment with anti-inflammatory compounds (data not shown). However, in our *in vitro* model of primary cultures of rat astrocytes triggered with gp120, we detected attenuation of both ERK1/2 and JNKs in the presence of minocycline, chloroquine and simvastatin (data not shown). In order to further demonstrate that the gp120-mediated activation of these pathways can be attenuated in our model, inhibitors of ERK1/2 (U0126) and JNK (SP600125) pathways were administered ICV daily prior to gp120 administration and we confirmed significant inhibition of ERK1/2 and JNK phosphorylation, respectively (Figure 7C and D).

## Discussion

A number of pro-inflammatory cytokines (TNF-α, IL-1β, IL-6, IFN-γ) and oxidative stress markers (such as nitric oxide) are known to be elevated during brain HIV-1 infection. HIV-1 coat protein gp120 has previously been shown to generate an inflammatory response and oxidative stress both *in vitro* and *in vivo *[3,5,23]. This viral protein is shed during HIV-1 replication and circulating levels of gp120 have been detected in plasma and in the CSF [31-35]. Our laboratory has extensively investigated gp120-mediated release of pro-inflammatory cytokines and oxidative stress in primary cultures of rodent astrocytes as well as in human fetal astrocytes exposed to viral isolates *in vitro *[3,5,6]. In this study, we implemented an *in vivo* model of brain inflammation by ICV administration of gp120. In different models, doses of gp120 ranging from 100 to 500 ng (administered intracerebrally) have been shown to induce an inflammatory response [29]. In our model, ICV administration of 500 ng of HIV-1 gp120_ADA_ resulted in a significant upregulation of different inflammatory markers in the frontal cortex, hippocampus and striatum. Heat-denatured gp120 failed to generate an inflammatory response, suggesting that a native gp120 structure is necessary to interact with chemokine receptors to induce an inflammatory response. We observed upregulation of IL-1β, iNOS and TNF-α in the frontal cortex of gp120-administered animals. In the hippocampus and striatum, IL-1β and iNOS transcripts were found to be elevated due to gp120 treatment. These markers are known to be upregulated during HIV-1 infection [4,28]. We also observed enhanced secretion of cytokines IL-1β and TNF-α in the CSF. Previously, increased GFAP expression has been observed in HIV-1 infected brain [36]. In our model, we observed a significant increase in GFAP expression in the hippocampus, indicating activation of astrocytes in this region.

Interaction of gp120 and chemokine receptors has previously been reported *in vitro *[5,6]. Previous work from our laboratory has detected expression of CCR5 in both human and rat primary culture of astrocytes. Using neutralizing antibodies against CCR5 and CXCR4 receptors, we have demonstrated that R5-tropic gp120 can interact with CCR5 chemokine receptors and result in pro-inflammatory cytokines (IL-1β, IL-6 and TNFα) secretion *in vitro *[5,6]. Maraviroc is a potent and specific inhibitor of CCR5 that is clinically used to treat HIV-1 infection. Consistent with our *in vitro* findings, maraviroc attenuated the gp120-mediated increase in IL-1β, iNOS and TNF-α in our *in vivo* model suggesting that the mechanism of gp120-associated inflammation is CCR5-receptor dependent. These findings provide evidence of *in vivo* interaction of gp120 with CCR5 receptor and emphasize the critical role of this interaction in generating a brain inflammatory response.

Downregulation of tight junction proteins (such as occludin, ZO-1, claudin5) has been implicated in the pathogenesis of brain HIV-1 infection [11]. A compromised blood-brain barrier can facilitate entry of HIV-1 from the periphery, and can ultimately increase the spread of the virus in brain parenchymal cellular compartments. Although *in vitro* and *in vivo* studies have demonstrated that HIV-1 proteins (like gp120 and tat) can downregulate tight junction proteins expressed in brain microvessel endothelial cells, [7,37], we did not observe any significant change in tight junction proteins expression in our model. This lack of effect may be due to the dose, duration and/or strain of the gp120 treatment we used. Persidsky *et al.* reported that downregulation of tight junction proteins is associated with Rho activation in brain microvessel endothelial cells and this activation was mediated by monocyte migration across these cells [38]. Ricardo-Dukelow *et al.* demonstrated that HIV-1 infected macrophages upregulate a number of proteins in brain microvessel endothelial cells that may lead to blood-brain barrier disruption [39]. Therefore, lack of immune cell infiltration could be another potential reason for lack of change in tight junction protein expressions in our model. We also did not detect any significant changes in transporter protein expression in the capillaries.

Tight junction proteins form a physiological barrier at the brain capillary level, whereas a biochemical barrier also exists both at the blood-brain barrier level and in brain parenchyma. This biochemical barrier primarily consists in the expression of drug efflux transporters and several metabolizing enzymes that restrict the bioavailability of a number of antiretroviral drugs into the brain. Modulation of these transporters by pathological stimuli has been previously reported [3,5,40,41]. Decreased P-gp expression at the gene and protein level has been reported in brain autopsy samples from patients with HIVE as well as in brain tissue collected from a severe combined immunodeficiency mice model of HIVE [11]. *In vitro,* HIV-1 viral isolates or gp120-mediated downregulation of P-gp has also been reported by our laboratory in both rodent and human astrocytes [5,6]. Consistent with these data, in our *in vivo* model, we observed downregulation of this transport protein expression in the frontal cortex and hippocampus further confirming that gp120 has a down-regulatory effect on P-gp functional expression in brain parenchyma.

Altered functional expression of Mrp1 has also been reported in the context of HIV-1. Hayashi *et al.* reported tat-induced upregulation of Mrp1, both at the gene and protein level in cultured astrocytes [40]. In primary cultures of rat astrocytes triggered with gp120 or TNF-α, we also detected an increase in Mrp1 functional expression [3,12]. In the present study, a significant upregulation of Mrp1 in the hippocampus and striatum was detected, further suggesting that gp120 exerts an up-regulatory effect on Mrp1 protein expression *in vivo*. In our hands, Bcrp was also found to be upregulated in the frontal cortex of gp120-treated animals. In contrast, IL-1β and TNF-α-mediated downregulation of Abcg2 has previously been reported in porcine brain microvessel endothelial cells [42]. These results indicate that, similar to other drug efflux transporters, Bcrp is susceptible to modulation by inflammatory cytokines. However, cytokine-mediated regulation of this transporter could be species and/or cell specific. Our *in vivo* data are consistent with previous *in vitro* findings in glial cells where exposure to gp120 resulted in a significant production of pro-inflammatory cytokines along with a downregulation of P-gp expression and an upregulation of Mrp1 expression [3,5,6]. Together, these observations provide evidence that regulation of drug efflux transporters is highly complex during HIV-associated brain inflammation. Since a number of antiretroviral drugs are known substrates for these transporters, changes in the expression of these transporters in brain parenchyma may result in an altered distribution of antiretroviral drugs at cellular targets of HIV-1 infection.

To date, only a few studies have reported on the neuroprotective and anti-inflammatory potential of different compounds (such as minocycline, curcumin, neurosteroids) in attenuating a HIV-associated inflammatory response [15,43,44]. The limitations of currently used antiretroviral drugs include lack of anti-inflammatory properties, poor brain permeability and neurotoxicity associated with better permeable drugs. Since neurological disorders are becoming more prevalent in HIV-1 infected patients and inflammation is a common immune response, identifying therapeutic compounds that can effectively permeate the blood-brain barrier, exhibit anti-inflammatory properties, and are well tolerated may provide an additional option in preventing and/or treating HIV-associated neurological disorders. For example, in a recent study, Maingat *et al.* reported efficacy of neurosteroid, dehydroepiandrosterone sulfate, in preventing inflammation and neurodegeneration as well as behavioral deficits in a feline immunodeficiency virus model [44]. Minocycline, a second generation tetracycline derivative, has been reported by many to have anti-inflammatory properties both *in vitro* and *in vivo *[15,45]. In an amyloid-beta injected rat model, minocycline has shown to successfully reverse the activation of glial cells [45]. In our study, minocycline also effectively inhibited gp120-mediated upregulation of different inflammatory markers in the frontal cortex, hippocampus and striatum and further prevented secretion of cytokines in the CSF. Recently, minocycline has been tested in a clinical trial in HIV-infected patients with cognitive impairment. Although this study reported that minocycline treatment was unsuccessful in improving neurocognitive function in patients with cognitive impairment, [21] it is still inconclusive if administration of the drug at earlier stages of the infection could have a better clinical outcome. Since minocycline has been reported to have a safe profile when administered with antiretroviral drugs in HIV-infected individuals, it will be important to assess if early administration of antiretroviral drugs along with minocycline has any beneficial effects against HIV-associated neurocognitive disorders. Several clinical studies indicate earlier administration of antiretroviral drugs can decrease the incidence of neurological disorders [46]. In a SIV model, early administration of minocycline was reported to be effective against striatal dopaminergic system dysfunction, suggesting that timely treatment initiation may have an effect on minocycline efficacy [15]. Since antiretroviral drugs (with the exception of maraviroc) do not have anti-inflammatory properties, a combination therapy including antiretroviral and anti-inflammatory compounds may prevent the development of HIV-associated cognitive deficits.

We also tested chloroquine, an antimalarial drug which exhibits anti-inflammatory and anti-HIV effects *in vitro* and *in vivo*. For example, chloroquine administration has been reported to reduce T-cell activation in chronic HIV-infected patients [47]. In a rodent model, chloroquine prevented a bacterial toxin-induced intracerebral toxicity [17]. In our gp120-administered model, chloroquine was able to suppress IL-1β and iNOS mRNA levels in the frontal cortex, hippocampus and striatum. Chloroquine was also effective in reversing gp120-mediated IL-1β release in the CSF. However, chloroquine was not able to inhibit gp120-mediated release of TNF-α. Clinical trials are necessary to determine whether chloroquine could be beneficial in reducing HIV-associated cognitive impairments, particularly in populations where malaria is also prevalent. Simvastatin, an HMG-CoA reductase inhibitor, is another compound that has demonstrated anti-inflammatory properties and neuroprotective effects [16,48]. In a study by Andras *et al.*, simvastatin prevented amyloid-beta and HIV-1 tat-induced upregulation of inflammatory genes in human brain microvessel endothelial cells [16]. In another study, this drug attenuated oxidative stress and inflammation and also restored short-term and long-term memory in a transgenic mice model of Alzheimer’s disease [48]. In our study, simvastatin was able to suppress IL-1β and iNOS in the hippocampus and striatum, but had minimal effect on IL-1β and TNF-α transcript levels in the frontal cortex. Also, the 1 mg/kg dose of simvastatin was not able to attenuate the secretion of cytokines in the CSF. In contrast to several other statins, simvastatin is known to have good permeability into the brain. However, in the periphery, simvastatin is highly metabolized by cytochrome P450 enzymes which can result in adverse drug-drug interaction with HIV-protease inhibitors making it a less ideal candidate for adjuvant therapy in HIV-infected individuals. Anti-inflammatory properties of statins that are not substrate of cytochrome p450 (rosuvastatin or pravastatin) or less potent inhibitors of cytochrome p450 (atorvastatin) should be considered in future studies.

Experimental data from various *in vitro* models suggest the involvement of different signal transduction pathways in the regulation of cytokine secretion and drug transporters during HIV-associated inflammation [6,12,40]. Potential anti-inflammatory compounds are also known to target different signaling pathways to attenuate the inflammatory response. In particular, MAPKs are widely known to be involved in various pathological processes associated with HIV-1 infection (such as neurotoxicity, macrophage activation, viral replication) [49]. Studies have further demonstrated that inhibition of these kinases can downregulate HIV-1 infection as well as decrease transcellular transport of HIV-1 across blood-brain barrier *in vitro *[49-51]. Therefore, we examined these pathways in our gp120-associated brain inflammation model and observed activation of ERK1/2 and JNKs in the hippocampus. The presence of minocycline or simvastatin decreased the gp120-mediated phosphorylation of ERK1/2 but not JNKs in the hippocampus. Since a number of studies suggest that chloroquine, simvastatin and minocycline can attenuate JNK activation *in vitro* or *in vivo*, we speculate that the lack of effect of these compounds on JNK phosphorylation could be time-dependent and could not be delineated in our *in vivo* model [20]. However, using specific inhibitors of ERK1/2 and JNK pathway, we were able to attenuate the gp120-mediated activation of these two pathways *in vivo*.

In summary, using a gp120-induced brain inflammation model *in vivo*, we have demonstrated that compounds with anti-inflammatory properties have the potential to prevent or reduce brain inflammation by interacting with signaling pathways. In our model, we have also observed altered expression of drug transporters due to gp120-mediated brain inflammation *in vivo.* The expression of ABC transporters expressed at the blood–brain barrier, in part, regulate the permeability of antiretroviral drugs into the brain. In addition, drug transporters localized at brain parenchymal cellular compartments also contribute towards the drug disposition in central nervous system. Alteration of these transporters due to HIV-associated pathogenesis and/or antiretroviral therapy may change the therapeutic outcome. Many different factors determine the effectiveness of antiretroviral therapy in improving neurocognitive impairment. We propose that identification of compounds with anti-inflammatory properties will significantly benefit patients with neurocognitive impairments.

## Conclusions

The major findings from our work revealed that agents such as minocycline, chloroquine and simvastatin which are known to display anti-inflammatory properties, are effective in reducing brain inflammation triggered by the HIV-1 viral envelope protein, gp120 in an *in vivo* rodent model. Furthermore, we demonstrated that this effect is mediated, in part, through an interaction with the MAPK signaling pathway. Future studies are needed to determine the efficacy of the early administration of antiretroviral drugs along with adjuvant therapy against the progression of HIV-associated brain inflammation as well as cognitive deficits.

## Abbreviations

ABC: ATP-binding Cassette; ANOVA: Analysis of variance; BCRP: Breast cancer resistance protein; CSF: Cerebrospinal fluid; ELISA: Enzyme linked immunoabsorbant assay; ERK1/2: Extracellular signal-related kinase 1/2; GFAP: Glial fibrillary acidic protein; gp120: Glycoprotein120; HAART: Highly active antiretroviral therapy; HAD: HIV-associated dementia; HAND: HIV-associated neurocognitive disorders; HIV-1: Human immunodeficiency virus-1; HIVE: HIV-encephalitis; HRP: Horseradish peroxidase; ICV: Intracerebroventricular; IFNα: Interferon α; IL-1β: Interleukin-1β; iNOS: Inducible nitric oxide synthase; JNKs: c-Jun N-terminal kinases; MAPK: Mitogen-activated protein kinase; MRPs: Multidrug resistance proteins; OCT: Optimum cutting temperature; P38Ks: P38 kinases; PBS: Phosphate-buffered saline; P-gp: P-glycoprotein; TNF-α: Tumor necrosis factor-α.

## Competing interests

The authors declare that they have no competing interests.

## Authors’ contributions

TA, WJ and RB designed the experiments. TA, WJ, MTH, JH and CW performed the experiments. TA, MTH and RB prepared the manuscript. All authors read and approved the final manuscript.
